# Urban Sloths: Public Knowledge, Opinions, and Interactions

**DOI:** 10.3390/ani8060090

**Published:** 2018-06-08

**Authors:** Kissia Ferreira Pereira, Robert John Young, Vanner Boere, Ita de Oliveira e Silva

**Affiliations:** 1 Department of Biological Sciences, Federal University of Viçosa, Viçosa 36570-900, Brazil; kissiakiu@yahoo.com.br; 2 School of Environment and Life Sciences, University of Salford Manchester, Salford M5 4WT, UK; r.j.young@salford.ac.uk; 3 Institute of Humanities, Arts and Sciences (IHAC), Federal University of Southern Bahia, Itabuna 45613-204, Brazil; itabio@hotmail.com

**Keywords:** brown-throated sloth, human-animal interactions, questionnaire, urban wildlife, *Bradypus variegatus*

## Abstract

**Simple Summary:**

Free-range sloths living in an urban environment are rare. In this study, opinions, attitudes, and interactions with a population of *Bradypus variegatus* were investigated through short, structured interviews and informal, opportunistic observations of people in the pubic square where the sloths live. A questionnaire was applied to people in the square where the sloths live. Opinions about population size differed greatly and younger people were concerned as to whether the square was an appropriate place for them. Some human-sloth interactions showed the consequences of a lack of biological knowledge. Sloths are strictly folivorous and are independent of human sources of food. Apparently, sloths are indifferent to humans. Despite the good intentions of people, there are many misconceptions about the behaviour and needs of sloths, which causes low wellbeing for the animals. These results demonstrate that actions in environmental education of the public could be beneficial for sloths.

**Abstract:**

Free-range sloths living in an urban environment are rare. In this study, the opinions, attitudes, and interactions with a population of *Bradypus variegatus* were investigated through short, structured interviews of people in the pubic square where the sloths live, in addition to informal, opportunistic observations of human-sloth interactions. A questionnaire was applied to people in the square where the sloths reside, and informal, opportunistic observations of human-sloth interactions were made. 95% of respondents knew of the sloths’ existence in the square and 87.8% liked their presence. Opinions about population size differed greatly and younger people were concerned as to whether the square was an appropriate place for them. Some human-sloth interactions showed the consequences of a lack of biological knowledge. People initiated all sloth-human interactions. The fact that sloths are strictly folivorous has avoided interactions with humans and, consequently, mitigated any negative impacts of the human-animal interaction on their wellbeing. These results demonstrate that, while there is a harmonious relationship between people and sloths, actions in environmental education of the square’s public could be beneficial for the sloths.

## 1. Introduction

There have been many studies concerning urban birds [1] and, more recently, urban mammals in the Neotropical region have started to receive the attention of researchers [2]. These urban mammals have included urban adapters or exploiters, such as marmosets [3] and opossums [4]. Generally, the mammal species found are small and have flexible behaviours [5]. However, some unexpected species, such as large obligate folivores, may be found within the urban environments of the Neotropics, including sloths. The presence of such species may result from direct human intervention (e.g., translocation) or because their habitat was engulfed by urban sprawl.

Although it can happen naturally, habitat fragmentation is one of the most prominent effects of environmental degradation caused by humans. Consequently, the reduction of natural habitats of wild animals increases the interaction between them and humans [6]. The presence of wild animals in urban environments seems to have a high aesthetic value [6,7] and was a probable reason for the practice of introducing wild animals to city squares in the past. However, the lack of knowledge about the biology of wild animals could lead people to adopt inappropriate behaviours, which could harm the wellbeing of the species involved. The most affected species appear to be those who are most likely to live in human crowded spaces [3]. In addition, it is believed that wild animals living in the urban environment have a greater availability of food resources and few natural predators [6].

Animal species, which arouse interest and promote physical contact, are considered calming agents [8]. Pets, for example, benefit the physical and mental well-being of humans, as is demonstrated in the treatment of depression and low self-esteem [9]. When investigating physical contact interactions between wild animals and humans, much research has focused on zoonotic diseases [10]. To date, few studies have examined how these interactions take place or humans’ perceptions of these interactions [3,11].

The brown-throated sloth (*Bradypus variegatus*) is a three-toed sloth belonging to a primitive group of mammals from the Pilosa order. Sloths are imperfect homeotherms because they cannot maintain a constant body temperature [12] and have a low metabolism, which is a consequence of their strictly folivorous diet. The low metabolism gives rise to the popular name of the species as they are slow in their movements and spend much of the day resting and sleeping [13]. Sloths spend most of their time in the trees (breeding, feeding, etc.). However, to defecate and urinate they descend to the ground, once or twice a week, and dig a hole with their tail, while the front legs are attached to the trunk of the tree [13].

Tiradentes Square in Teófilo Otoni, Brazil, has had a population of brown-throated sloths in a highly urbanized area since, at least, the 1960s. These animals are isolated from their natural environment and frequently interact with the city's human population. The aims of this research were to: (1) To verify that the human population knows about the sloths in the square; (2) to investigate their views regarding the presence of these animals in an urban area; and (3) to describe human-sloth interactions. This research was done by conducting interviews using a structured questionnaire and through informal observations of human-sloth interactions.

## 2. Materials and Methods

### 2.1. Study Area

The study was conducted in Tiradentes Square in Teofilo Otoni, Minas Gerais, Brazil (17°51′52.73″ S, 41°30′29.48″ W). Teofilo Otoni is a city in the northeast of Minas Gerais, located in the Mucuri River Valley, and has a population estimated at 140,000 inhabitants [14]. Currently, the city borders contain an area of 3242 square kilometers. The climate is tropical with an average annual temperature of 23 °C; it has dry, mild winters and rainy summers with high temperatures [14].

Tiradentes Square is the main square of Teofilo Otoni and the city’s main traffic artery runs through the square. The square is widely used by the population for trade, especially Aquamarine stone; it also contains bus stops for all municipal lines, as well as some inter-municipal lines, which makes the square heavily visited.

In the square, there is a population of brown-throated sloths (seven adult males, one adult female, and one infant of an undetermined sex). Manchester and Jorge [15] recorded 85 trees in the square of various species. Of these, only nine (10.58%) are a species (*Ficus* spp.) that is a natural food source for sloths. The other 76 trees are of species commonly used as ornamentation in cities 

### 2.2. Interviews

Interviews were carried out using a structured questionnaire targeting people passing through Tiradentes Square in Teofilo Otoni, Minas Gerais, between the 18 and 27 of December 2014. One hundred and eighty-two people (91 female and 91 male) were interviewed by the researcher (Table 1). The interviews took place from 08:00 to 12:00 hours and from 14:00 to 17:00 hours. The researcher randomly approached people who were already stationary to avoid disrupting the daily lives of passers-by. The questions were applied by the researcher and, thus, any doubts could be immediately clarified.

The interview was comprised of 13 questions, five demographic factors (gender, age, occupation, how long the participant has been a resident in the city, and the frequency of visits to the square). Three questions asked about the participant’s knowledge of the sloths in the square (did they know there were sloths, had they seen them, how many sloths did they think lived in the square). Two questions investigated participants’ general knowledge of sloth biology (what sloths eat, do sloths come to the ground). Finally, three questions identified the participant’s perceptions about having these wild animals in urban areas (do they think the sloths are healthy, did they like to see sloths in the square, and what should be done with the sloths living in the square).

This study was approved by the Human Ethics Committee (CAAE: 32229514.4.0000.5153, Filed on: 23 September 2014) of the University of Viçosa, Minas Gerais, Brazil.

### 2.3. Data Analysis

We used Minitab 16.0 for Windows for descriptive analysis of the data: From the data we used the percentage of valid answers to each question. To analyse the effects of age and sex on some of the questions presented, we analyzed the frequencies of responses using chi-squared tests and alpha <0.05.

### 2.4. Observations of Human-Sloth Interactions

*Ad libitum* observations of the interactions between humans and sloths were undertaken. The aim of these observations was to classify the types of human-sloth interactions spontaneously occurring in the square.

## 3. Results

### 3.1. Questionnaire: Social Demographics

The questionnaire sought to create a demographic profile of the respondents. The mean age of respondents was 40 years (±16.6), with the youngest aged 13 and the oldest aged 84 years. Of the respondents, 67% lived in Teofilo Otoni and 50% had been a resident for over 20 years. Many of the respondents (43.3%) frequented the square more than five days a week, while 28.3% frequented the square at least two to three times a month.

### 3.2. Sloth Existence in the Square

Most respondents (95%) knew of the existence of sloths in Tiradentes Square and 90.6% had seen them in the trees. The majority (56.5%) reported that no more than five sloths resided in the square, 25.4% believed there were up to 10 sloths, and 4% said there were more than 20 sloths. In contrast, 6.8% of respondents thought that sloths no longer lived in the square.

### 3.3. Sloth Biology

In relation to diet, 69.5% correctly answered that sloths feed only on leaves, 23% said that they also eat fruits, 2.3% answered leaves, fruits, and meat, and 5.2% believed they feed on any food that is offered.

Twenty-four percent of respondents believed that sloths never go down to the ground, 19.6% believed that sloths go down to the ground to look for food, 32.4% said that they go down to defecate, and 24% could not say why sloths would go down to the ground, but believed that they do. When comparing these two questions, we observed that 40% of respondents who answered the first question correctly also got the second question correct.

### 3.4. People’s Perceptions about Sloths

Sixty-nine percent said they believed that the sloths were healthy, and this is one of the reasons that 87.8% liked to see them in the square. Only 3.3% did not like to see them in the square and 8.8% liked to see them sometimes. Of the 22 people who said they did not like or sometimes liked to see these animals in the square, 17 believed that animals are not healthy. 

**Figure 1:**
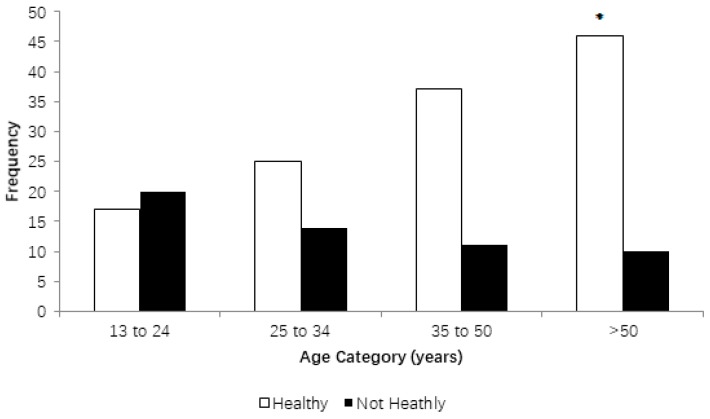
Relationship between the ages of respondents who thought that the brown-throated sloths in Tiradentes Square, Teofilo Otoni, Minas Gerais, Brazil were healthy. * *p* < 0.05.

Gender had no significant effect on whether people thought the sloths were healthy or not (*p* > 0.05). Though there was no effect of gender (*p* > 0.05), it was found that people over 50 were significantly more likely to believe that the sloths were healthy than the younger age groups (χ^2^ = 15.72; DF = 3; *p* < 0.001) (Figure 1).

**Figure 2:**
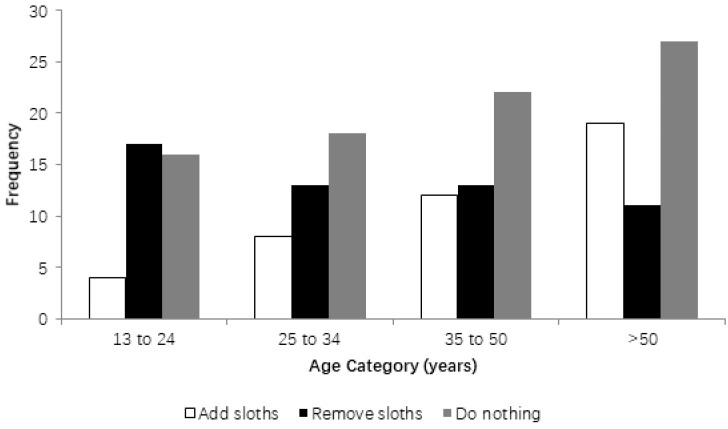
Relationship between the ages of respondents and their opinions about what should be done with the brown-throated sloths in Tiradentes Square, Teofilo Otoni, Minas Gerais, Brazil.

Respondents were asked about what should be done with sloths living in the square: 23.9% thought more sloths should be added; 30% thought that the sloths must be translocated to another location, such as a forest; and 46.1% thought that the animals should continue in the square without any interference in the population size. There was no significant difference in the preference of older or younger people in terms of what should be done with the sloths (*p* > 0.05) nor a difference by gender (*p* > 0.05) (Figure 2).

### 3.5. Informal Observations of Human-Sloth Interactions

The types of interaction observed were: Look for sloths, talk to them, take photographs, hold sloth in arms, remove sloth from its location, and offer food to sloth. All observed interactions were initiated by people; however, there was no reciprocity on the part of the animals for any type of interaction.

## 4. Discussion

### 4.1. Questionnaires

**Figure 3:**
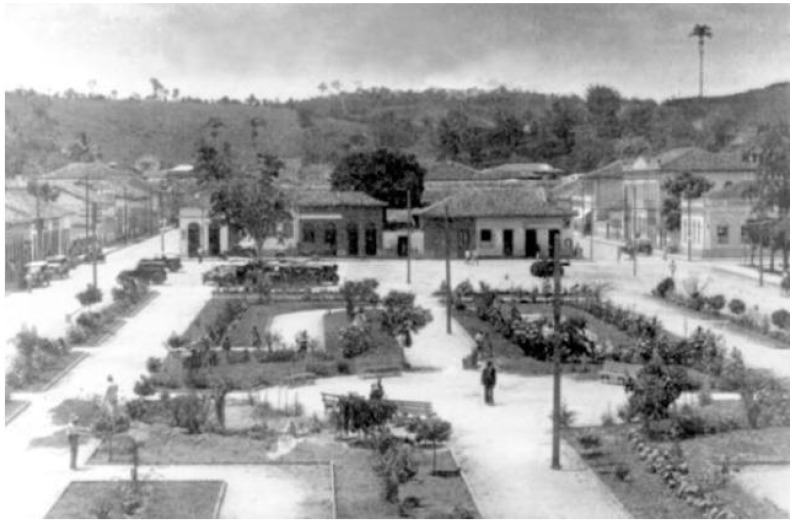

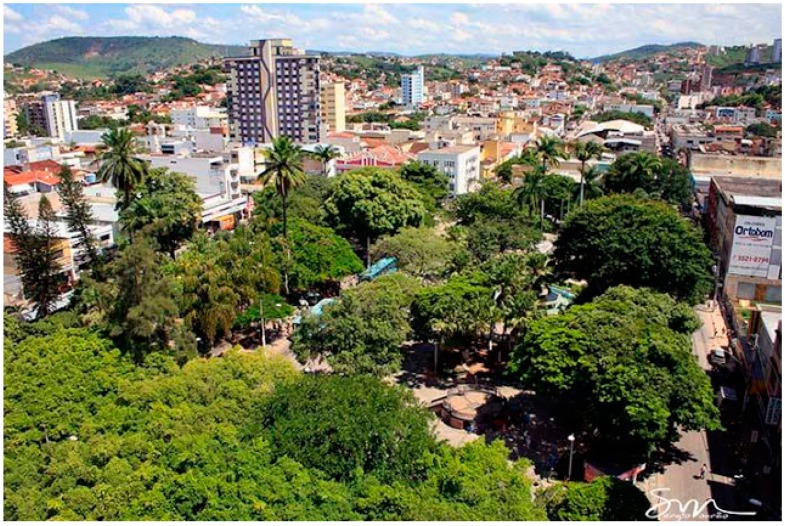
Vintage photograph of the Tiradentes Square, *circa* 1930, compared to a recent photograph. The vintage is freely available at the Teófilo Otoni municipal administration internet site (http://www.teofilootoni.mg.gov.br/site/sobre/historia/, copy at 10 December 2017). The recent photograph is authored by Sérgio Mourão, and freely available in http://www.conhecaminas.com/2017/05/15-lindas-e-charmosas-pracas-de-minas.html, 27 May 2018.

In the questionnaires, we can see that most respondents have seen and knew of the existence of the sloths in the square. Of the nine respondents who said they did not know of the existence of these animals in the square, only one was a resident of Teófilo Otoni and of those who have never seen the animals, only four lived in the city. Older residents reported the existence of hundreds of animals in the square and said that they were there since the creation of the city on 7 September 1853. Other residents, however, suggested that the sloths were placed in the square. This second hypothesis is more probable, since old photographs show the square without any trees (Figure 3). However, the number of sloths has been much higher, and the population has declined significantly in recent times, from 25 in the year 2008 [15] to eight in the present study. Most respondents believed that there were few animals in the square and were surprised when they were informed of the number of animals. Of the people who said there were more than 20 sloths 50% said had not perceived the decline in population. Several people suggested that many sloths were stolen and sold to tourists, however, this information has never been proven. Teófilo Otoni is on the route of wildlife trafficking in southeast Brazil, although it is not considered by RENCTAS (National Network for Combating Trafficking of Wild Animals) as a local capture point for animal trafficking [16]. Latin America provides for the pet trade and ‘transforms’ wild animals into pets [17]. Thus, the most trafficked species are those that appeal to human caregiving instincts and, thereby, engender affection and empathy in people [18,19] and, consequently, a desire for ownership. Sloths fall into this category and are a target of this illegal trade. However, our study demonstrates evidence that sloths are a cultural patrimony of the city and that robbery is unlikely due the effects on the popular reaction. Unfortunately, the lack of information in relation to sloth trafficking and sloth mortality makes it difficult to know the reason for the population’s decline.

Sloths are an attraction in the square, especially for children and tourists. It is common to see people looking at the trees searching for animals and when they descend to the ground people approach them to take pictures and to pet them. Most respondents knew that sloths eat leaves; many people said only leaves because they realized that the trees in the square, mostly, did not produce fruit. Some respondents said they had tried to give other foods (such as savoury items and bananas), but the animals did not accept these. Vining [18] states that the fact that people try to feed animals is a way to get into the animal’s world and, thus, connect with nature. Empathy to non-human animals is widespread across cultures and, particularly, well studied in occidental, western civilization (e.g., [20]). Empathy may be a major reason why people try to make physical contact with the sloths, even though the animals show no reciprocity and respond by ignoring, avoiding, or trying to escape from people. These responses suggest that uninvited and, essentially, forced interaction with people is a source of distress and could be potentially detrimental to their welfare, as in other species (e.g., [21,22]). For the sloths, this direct contact with humans is limited by their status as folivores and this has, no doubt, been essential for their overall health and welfare.

The sloths have been in the square for a long time, and people have direct contact with them. Despite this, the lack of knowledge on sloth biology was surprising. The three-toed sloths only defecate in soil, preferably in a place with soft earth where they can dig a hole with their tail. However, this information was unknown by most respondents when asked if sloths go down to the ground and for what purpose, with the responses being quite varied. This lack of knowledge often leads to inappropriate behaviour of the square visitors who, when faced with the sloths on the ground, put them back in the trees, believing this to be a risky behaviour for sloths. Thus, the animals need to come down again to the ground to defecate, which increases their energy expenditure. Another situation observed was sloths using the ground to move to another tree, but failing to reach its destination because people put them in the same tree from where the animal had just come from.

Many people believed that the sloths were healthy and this conclusion was derived from the fact that the animals continue to survive and breed in the square. Despite the number of sloths having decreased considerably, in recent years, people have not associated this with health problems. When we analyzed the answers concerning the health of the sloths by respondents’ gender, we found that 62% of women and 77% of men thought that they were healthy. Although this difference was not significant, previous studies [23] have shown that women have a greater concern for the welfare of animals in relation to men.

Such types of relationships between humans and animals are usually made to bring perceived benefits to both species, such as the human finding that the animal acts as a calming agent [8,24], though this may not always be the case for the animal (e.g., [22]). There was no significant difference between the percentage of men and women who like to see these animals (87% of men and 89% women). Most of those who did not like or sometimes liked to see sloths in the square believed that they were not healthy (77.27%). The fact that older people (82%) believed that the animals were healthier than the younger people (46%), shows that they have different perceptions of animal wellbeing. Studies have shown that older people express less care or concern for animals than younger people [25], though this may reflect cultural, knowledge, and educational changes rather than an age effect *per se*.

When asked what they think should be done with the sloths from Tiradentes Square, people thought it was best to leave things as they were because sloths were already adapted to the environment, despite the noise and air pollution, and believed that to take them to another place would be bad for the sloths’ wellbeing. Additionally, people believed that more animals should not be introduced to the square as it is not the ideal environment for them. Those who thought they should bring more animals into the square would like to restore the “original” and larger sloth population size because they believed that the population decrease was mainly due to traffic. These responses were, especially, given by older people who had, historically, witnessed a larger population living in the square. Compared to young and middle-aged people, the thoughts of old people are regularly referenced to the past and show less concern about the future [26]. 

Those in favour of removing sloths from the square argued that they should be taken to a forest or a reserve so that they could be in their natural environment; this response was given mainly by the younger respondents. Young people were more conscious about the relationships between nature and urban life, the right place for wild animals, and ecological concepts, since ecology is, currently, a matter of Brazilian formal teaching [27]. Higher education is correlated to more knowledge about wild animals [28]. In fact, formal education access was increased over the past decade in Brazil and older people did not have the same access to a higher-level education. Therefore, the influence of the media, television, movies, and the internet is motivating young people to build a new *constructo* of morality to wild animals, nature, and biodiversity [29] compared to older ones. Despite this positive aspect, young people had an idealistic and romantic view about the right place for sloths, a wild animal species. This group thought that the forest would be an ideal place, despite the logistic troubles and the health and wellbeing of the sloths, which are ignored in a putative translocation program [30]. The morality of this idealization encompasses the symbolic world of an untouchable nature and portrays sloths as things, which are part of this pure world. Thus, sloths in the Tiradentes square are a part of the symbolic world about nature to the young and old people of Teofilo Otoni city. We think that sloths in the Tiradentes square are a cultural symbol to the Teofilo Otoni people and that this is more significant than the real needs of the animals. 

### 4.2. Ad Libitum Observations of Human-Sloth Interactions

People who frequented Tiradentes Square are already habituated to the presence of sloths and often did not pay attention to the fact that they were there unless the animals were near to the ground. When the animals were near the ground people tried to interact with them. The interactions for the most part were people trying to take pictures of these animals or trying to pet them. Additionally, we observed people touching, picking up, arising, talking to, throwing snack foods, and following the sloths. However, the sloths did not show any reaction to these interactions and acted as if people were not there. People of all ages and both sexes tried to interact with the sloths. Sometimes children tried to approach the sloths and their caregivers prevented them due to fear of the sloth hurting them due to their large claws. The sloths did not show any behavioral fear of people and sometimes crawled along the ground to change trees, crawling among people.

Some patrons of the square tried to feed the animals, but since they are strictly folivorous they did not accept any other type of food and this practice has decreased over time. People usually offer food to animals out of concern for animal welfare [31] because they believe that this habit is important and to also create a dependent relationship. However, sloths do not eat any food apart from the leaves of the *Ficus* tree. 

## 5. Conclusions

Our study evidences that sloths are a cultural patrimony of the city and it is suggested that robbery of the animals from the square causes a public reaction. However, such theft would be quite feasible. The sloth population has declined from 2009 [15] to eight animals in 2014. The lack of information about potential trafficking and about factors contributing to sloth mortality, such as human interaction and the few appropriate forage trees, makes it difficult to know the reason for the population’s decline. Environmental education of the square’s public could be beneficial for the sloths. Furthermore, the present population, containing only one female in this slow breeding species, is not sustainable in terms of maintaining sloths in the long-term in the square. Intervention will, eventually, be needed to prevent this group from dying out, but as the results show this should be done in consultation with the public.

## Figures and Tables

**Table 1:** Demographic characteristics of respondents interviewed in Teofilo Otoni, Minas Gerais, Brazil about the presence of brown-throated sloths in Tiradentes Square (we used the valid percentage as data, since some people did not answer all questions). T.O. = Teofilo Otoni.

Category	Frequency	Percentage (%)
**Sex**		
Male	91	50
Female	91	50
**Age**		
13 to 24 years	37	20
25 to 34 years	39	22
35 to 50 years	49	27
>50 years	57	31
**How long have you lived in T.O.?**		
I do not live in T.O.	60	33
<1 year	3	1.60
1 to 5 years	8	4.40
5 to 10 years	5	2.70
10 to 20 years	15	8.20
>20 years	91	50
**Frequency of square visits**		
2 times per month	49	28.30
1 time per week	26	15
3 times per week	23	13.30
5 times per week	21	12.10
Every day	54	31.20
